# Capsular Contracture in Implant-Based Breast Reconstruction: A Comprehensive Narrative Review of Pathophysiology, Risk Factors, and Contemporary Controversies

**DOI:** 10.3390/medicina62050831

**Published:** 2026-04-27

**Authors:** Mihai Iliescu-Glaja, Fabiana Simion, Dana Stoian, Daciana Grujic, Cristi Tarta, Razvan Bogdan, Zorin Crainiceanu, Teodora Hoinoiu, Andrei Motoc

**Affiliations:** 1 Doctoral School, “Victor Babes” University of Medicine and Pharmacy, E. Murgu Square, No. 2, 300041 Timisoara, Romania; mihai.iliescu.glaja@umft.ro (M.I.-G.); razvan.bogdan@umft.ro (R.B.); 2 Plastic and Reconstructive Surgery Department, Pius Brinzeu Clinical County Emergency Hospital Timisoara, Casa Austria, Liviu Rebreanu Blvd. 156, 300723 Timisoara, Romania; fbn.simion@gmail.com (F.S.); crainiceanu.zorin@umft.ro (Z.C.); tstoichitoiu@umft.ro (T.H.); 3 Department of Internal Medicine II, Discipline of Endocrinology, “Victor Babes” University of Medicine and Pharmacy, E. Murgu Square, No. 2, 300041 Timisoara, Romania; stoian.dana@umft.ro; 4 Plastic Surgery Department, “Victor Babes” University of Medicine and Pharmacy, E. Murgu Square, No. 2, 300041 Timisoara, Romania; 5 Center for Advanced Research in Cardiovascular Pathology and Hemostaseology, “Victor Babes” University of Medicine and Pharmacy, E. Murgu Square, No. 2, 300041 Timisoara, Romania; 6 Researching Future Surgery II Research Center, Department X, Discipline of General Surgery II, Faculty of Medicine, “Victor Babes” University of Medicine and Pharmacy, E. Murgu Square, No. 2, 300041 Timisoara, Romania; 7 Discipline of Clinical Skills, Department I Nursing, “Victor Babes” University of Medicine and Pharmacy, E. Murgu Square, No. 2, 300041 Timisoara, Romania; 8 Department of Anatomy and Embryology, Faculty of Medicine, “Victor Babes” University of Medicine and Pharmacy, E. Murgu Square, No. 2, 300041 Timisoara, Romania; amotoc@umft.ro

**Keywords:** capsular contracture, implant-based breast reconstruction, postmastectomy radiotherapy, acellular dermal matrix, polyurethane implants, prepectoral reconstruction, foreign body response, clinical decision framework, breast cancer, patient-reported outcomes

## Abstract

Capsular contracture (CC) remains the most common long-term complication of implant-based breast reconstruction (IBBR), significantly impacting cosmetic outcomes, patient satisfaction, and reoperation rates. Despite substantial advances in surgical technique, implant technology, and perioperative management, the incidence of clinically significant contracture persists at approximately 3–5% at five years in non-irradiated patients and escalates dramatically—to 20–50%—in those receiving postmastectomy radiation therapy (PMRT). The etiology is multifactorial, involving subclinical biofilm formation, a dysregulated host immune and foreign-body response, and radiation-induced fibrosis. This narrative review synthesizes contemporary evidence on the pathophysiology, clinical assessment, and modifiable risk factors for CC in IBBR, with particular emphasis on implant surface characteristics (smooth, textured, and polyurethane[PU]-coated), placement plane (prepectoral versus subpectoral), the role of acellular dermal matrices (ADMs), reconstruction timing (direct-to-implant versus two-stage), and the complex interplay with radiotherapy—including radiation timing, fractionation, and emerging delivery techniques. We also address ongoing controversies, including the lack of standardized objective diagnostic criteria, the comparative effectiveness of ADM versus PU-coated implants, and the optimal sequencing of radiation relative to reconstruction. By integrating the latest evidence from very recent major meta-analyses and national registries, this review provides an updated synthesis. We further propose an evidence-based clinical decision framework for CC risk mitigation. This review aims to inform individualized surgical decision-making and identify priority areas for future investigation.

## 1. Introduction

Implant-based breast reconstruction (IBBR) has become the predominant approach for restoring the breast after mastectomy, largely due to its relative simplicity and shorter recovery compared to autologous tissue reconstruction. In the United States, implant-based methods accounted for ~80% of all breast reconstructions as of recent national data, reflecting a steady increase in their use alongside advances in surgical techniques and implant technology [1,2]. When applied in appropriately selected patients—including immediate direct-to-implant (DTI) procedures—implant-based techniques can achieve excellent aesthetic outcomes with high patient satisfaction [1,2]. However, these benefits are tempered by the risk of prosthesis-related complications [1,2,3]. Among these, capsular contracture (CC) remains a leading and persistent issue, often undermining long-term reconstructive success.

CC is the pathological tightening of the fibrous capsule that normally forms around a breast implant. This excessive scar contraction can cause breast hardness, pain, distortion of breast shape, and implant malposition. It is one of the most common complications following IBBR and a major cause of reoperation [3,4]. Reported rates of CC vary widely—from roughly 2% to nearly 40% of cases, depending on follow-up duration, grading criteria, and patient population [3,4,5]. By contrast, exposure to post-mastectomy radiation therapy (PMRT) dramatically increases the risk of contracture: CC has been reported in roughly 20–50% of irradiated reconstructions, compared with approximately 3–5% of non-irradiated cases at five years [5,6,7]. Severe contracture contributes substantially to patient morbidity and frequently necessitates revision surgery or implant removal [7,8].

The etiology of CC is multifactorial, and numerous factors have been implicated in its development. Implant characteristics play a role: silicone gel implants may be associated with slightly higher rates of contracture than saline implants, while the influence of implant surface texturing remains debated in the reconstructive context [8,9]. Recent meta-analytic evidence suggests no significant difference in contracture rates between textured and smooth implants in reconstruction patients, though this parity may be confounded by concurrent use of acellular dermal matrices (ADMs) [9,10]. Surgical variables are also important: the implant placement plane (prepectoral versus subpectoral) remains an area of active investigation, with emerging data showing comparable or even lower contracture rates with prepectoral approaches using ADM support [11,12,13]. The role of ADMs in modulating periprosthetic fibrosis is another key modifiable factor, though high-level evidence for a definitive protective effect remains inconclusive, and routine use is balanced against cost and complications such as seroma—the most frequently reported ADM-associated complication [14,15,16].

Despite many advances, several controversies and knowledge gaps persist regarding CC in IBBR. It remains unclear whether two-stage reconstruction with a temporary tissue expander (TE—allowing radiation before implant exchange) is superior to an immediate DTI approach in reducing contracture, as studies have reported conflicting results [17,18,19]. Several studies suggest radiation is better tolerated on an expander, associated with lower overall complication rates, and any complications that arise affect a temporary device that will be exchanged. Although the permanent implant itself is not irradiated in this sequence, it is important to note that radiation-induced changes to the surrounding soft tissue—including microvascular injury, fibrosis, and altered cytokine milieu—persist after implant exchange and continue to influence the long-term reconstructive environment; CC that develops on the expander is addressed surgically at the time of exchange, but the underlying profibrotic tissue substrate remains [17,20]. However, this lower complication rate is also partly attributable to the shorter in situ time of the expander compared to a permanent implant [18]. The ideal implant placement plane in the setting of radiation is also unresolved. Furthermore, inconsistency in defining and grading CC—often relying on the subjective Baker classification—makes it challenging to compare outcomes across studies [21,22].

Given the prevalence of IBBR and the clinical burden of CC, an updated synthesis of this rapidly evolving evidence base is warranted. Several recent reviews have addressed individual aspects of CC in IBBR; however, the past two years have seen the publication of multiple large-scale meta-analyses and national registry studies that substantively reshape several key conclusions—including the equivalence of implant surface types in reconstruction [9,10], the convergence of CC rates across placement planes when ADM is accounted for [9,11,12,13], the quantified magnitude of radiation-associated risk [17,18], and the emergence of polyurethane(PU)-coated implants as a potential alternative to ADM-covered devices. The present narrative review integrates these recent findings—drawn from studies published through early 2026—into a comprehensive, clinically oriented framework. In addition to synthesizing the current evidence on pathophysiology, risk factors, and management, we propose an evidence-based clinical decision framework for CC risk mitigation in IBBR. By consolidating and contextualizing the most current data, this review seeks to inform individualized surgical decision-making and to identify priority areas for future prospective investigation.

## 2. Literature Search Strategy

This narrative review was conducted following an expert-guided approach to literature identification, selection, and synthesis. Although a structured search strategy was employed to ensure comprehensive literature coverage, this review was not conducted according to a predefined systematic protocol (e.g., PRISMA), and the search was not registered in a systematic review registry. The structured search methodology described below is intended to maximize the transparency and reproducibility of study identification, but it does not constitute a systematic review.

A comprehensive electronic search of PubMed/MEDLINE, Scopus, and the Cochrane Library was performed from January 2010 through January 2026, using the following search strategy: (“capsular contracture” OR “capsule fibrosis” OR “periprosthetic fibrosis”) AND (“breast reconstruction” OR “implant-based breast reconstruction” OR “tissue expander” OR “direct-to-implant” OR “prepectoral” OR “subpectoral”) AND (“implant” OR “prosthesis”). The terms ‘prepectoral’ and ‘subpectoral’ were incorporated directly into the primary search string rather than reserved as specific modifiers because implant placement plane constitutes one of the most actively investigated and clinically consequential variables in contemporary IBBR, and their inclusion at the primary level was intended to ensure comprehensive capture of relevant comparative studies without risk of omission through modifier-based filtering. Additional targeted searches were conducted combining these terms with specific modifiers, including “acellular dermal matrix”, “polyurethane” (PU), “radiotherapy,” “ postmastectomy radiation therapy,” “Baker classification,” “biofilm,” and “implant-associated anaplastic large-cell lymphoma” (BIA-ALCL). Only English-language publications were considered.

Inclusion criteria required studies to address CC incidence, pathophysiology, prevention, or management in the specific context of IBBR (DTI or two-stage expander/implant techniques). We prioritized high-level evidence, including recent meta-analyses, randomized controlled trials, national registry studies, and multicenter cohort studies published from 2010 onward, to comprehensively capture contemporary practice. Retrospective single-institution series were included when they provided unique comparative data not available from higher-level sources (e.g., PU vs. ADM comparisons, radiation timing cohorts). Studies focusing exclusively on cosmetic augmentation were excluded, except where augmentation-specific meta-analyses provided the only available evidence for a given comparison (e.g., implant surface or filling material); in such cases, the distinct clinical context was explicitly noted. Case reports, animal studies, and non-English publications were excluded.

Reference lists of all included meta-analyses and pivotal studies were manually screened to identify additional relevant sources. Selected evidence was grouped by thematic domains—pathophysiology, clinical assessment, implant characteristics, surgical technique (placement plane, ADM, reconstruction timing), radiotherapy interaction, and clinical outcomes—and synthesized to identify areas of established consensus versus ongoing controversies. Given the narrative design, no formal risk-of-bias assessment or quantitative pooling was performed—an inherent limitation of the narrative design that precludes formal quality appraisal of included studies; however, the level of evidence (meta-analysis, RCT, registry, retrospective cohort) is indicated throughout to allow the reader to gauge the strength of individual findings.

The electronic database search yielded 1486 records across all three databases. After removal of duplicates, 948 unique records underwent title and abstract screening, of which 237 proceeded to full-text review. Following application of inclusion and exclusion criteria, 74 studies formed the core evidence base. An additional 17 references were identified through manual screening of reference lists of included meta-analyses and pivotal studies, and 5 seminal pre-2010 works were retained for foundational and historical context, yielding a final total of 96 cited sources.

## 3. Pathophysiology of Capsular Contracture

**Figure 1:**
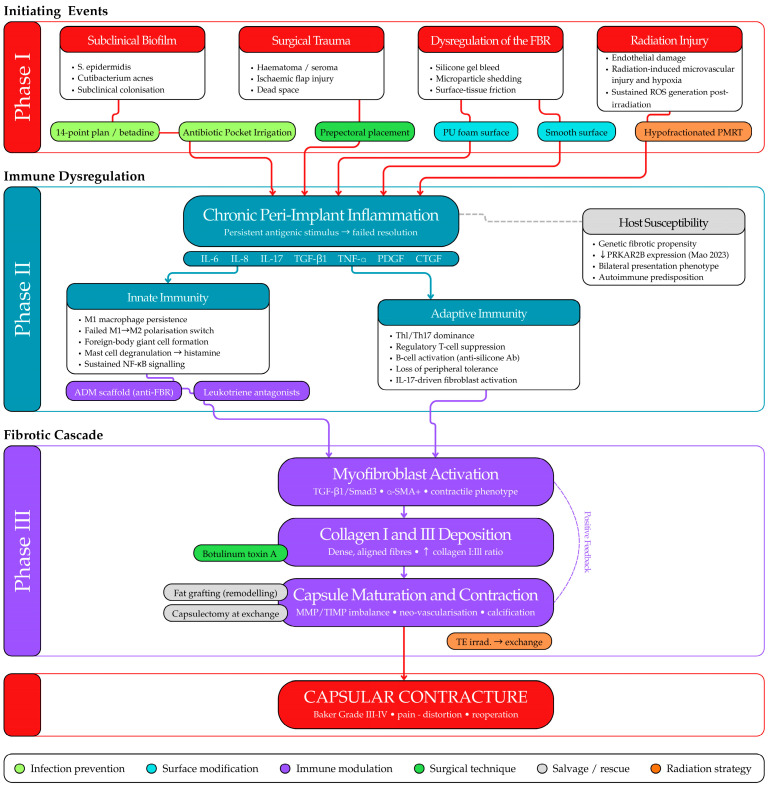
Pathogenesis of capsular contracture and points of therapeutic intervention. Four initiating events (Phase I, red)—subclinical biofilm, surgical trauma/hematoma, the foreign-body response to the implant, and radiation injury—converge on chronic peri-implant inflammation (Phase II, blue), which through innate and adaptive immune dysregulation (M1 macrophage polarization, Th17/Treg imbalance) drives myofibroblast activation and progressive collagen deposition (Phase III, purple), culminating in clinically significant contracture (red). Post-mastectomy radiotherapy amplifies all phases of the cascade. Current therapeutic interventions are mapped to their primary target mechanism. FBR = foreign body response; ROS = reactive oxygen species; α-SMA = alpha-smooth muscle actin; ADM = acellular dermal matrix; PU = polyurethane; Ab = antibody; IL = interleukin; TGF-β1 = transforming growth factor-beta 1; TNF-α = tumor necrosis factor-alpha; PDGF = platelet-derived growth factor; CTGF = connective tissue growth factor; NF-κB = nuclear factor kappa B; MMP = matrix metalloproteinase; TIMP = tissue inhibitor of metalloproteinases; TE = tissue expander; PMRT = postmastectomy radiation therapy; M1/M2 = classically/alternatively activated macrophage phenotypes; Th1/Th17 = T helper 1/17 cell subsets; Treg = regulatory T cell. N.B.: Illustrated by our graphic designer, Marius Filip.

CC is broadly understood as an excessive fibrotic response to the breast implant, driven by chronic inflammation in the peri-implant capsule. However, the triggering causes of this inflammatory cascade remain a subject of debate. Most experts agree that the etiology is multifactorial, with several major hypotheses proposed to explain why some reconstruction patients develop severe capsule fibrosis while others do not [3] (Figure 1).

### 3.1. The Bacterial Biofilm Hypothesis

One widely supported theory implicates subclinical bacterial infection and biofilm formation on the implant surface as a key inciting factor for CC. According to this hypothesis, skin flora introduced at the time of surgery (such as *Staphylococcus epidermidis* or *Cutibacterium acnes*) can attach to the implant and form a biofilm, evading host defenses and antibiotics. The biofilm induces a sustained low-grade immune response at the implant–tissue interface, leading to chronic inflammation and fibrosis of the capsule [23,24]. Culture-based analyses of capsules removed for contracture frequently grow *S. epidermidis*, *C. acnes*, or *Streptococci* [24]. In a microbiological study of 65 patients, Galdiero et al. found that all pathogenic bacteria and fungi persisting on the skin after povidone-iodine disinfection were biofilm producers, and that the same strains identified on the capsule and implant surfaces matched those detected on the corresponding skin and nipple-areola complex [25]. Capsular contracture was more frequent among oncologic patients who received radiotherapy, patients with prior contracture, and patients with cutaneous colonization by biofilm-producing organisms [25].

However, molecular studies employing PCR and next-generation sequencing have yielded a more nuanced picture. A 2019 study by Bachour et al. using a highly sensitive PCR assay (IS-pro) found that both normal and contracted breast capsules were generally sterile, with only trace amounts of *Staphylococcus* spp. detected in 4 of 50 capsules and no difference in bacterial presence between groups (*p* = 1.0); the authors concluded that the few organisms detected likely represented intraoperative contamination at removal rather than true capsular colonization [26].

A recent microbiome sequencing study by Park et al. (2025) found that contracted capsules showed a trend toward higher total bacterial load and greater relative abundance of *Staphylococcus* compared to non-contracted capsules, though neither difference reached statistical significance (*p* = 0.380 and *p* = 0.101, respectively) [27]. Total bacterial load correlated positively with *Staphylococcus* abundance (r = 0.610, *p* = 0.001), and source-tracking analysis indicated that the majority of capsular microbiota (>84%) originated from unknown—potentially endogenous—sources rather than from skin contamination [27]. Notably, capsular microbiome composition was more strongly influenced by individual patient factors (age, implant duration) than by contracture status [27]. Taken together, these molecular findings suggest that while bacteria are detectable in some capsules, their role may be more complex than straightforward exogenous contamination, and the contribution of endogenous breast flora warrants further investigation.

Despite these encouraging findings and the overall weight of supporting evidence, the biofilm hypothesis remains incompletely proven, and the interplay between bacterial load, biofilm formation, endogenous flora, and host immune response requires further clarification [24,25,26,27,28,29,30].

### 3.2. Non-Infectious Inflammation and Foreign Body Response

Another major theory posits that any source of persistent sterile inflammation around the implant can provoke the same pathologic fibrotic response. From this viewpoint, CC represents an exaggerated fibrotic healing response to a persistent foreign body stimulus—sharing histological features with hypertrophic scarring, such as myofibroblast activation and excess collagen deposition, but critically distinct in that the immune response to the implant itself, including macrophage polarization and T-cell dysregulation, plays a central and obligatory role that has no direct parallel in cutaneous hypertrophic scarring [23].

Even in the absence of overt infection, various non-microbial factors can sustain local inflammation and drive collagen deposition and myofibroblast activation within the capsule. Clinical observations strongly support this: known risk factors for CC include tissue injury and postoperative complications that elevate inflammatory cytokines. For example, an acute hematoma in the implant pocket is well known to predispose to contracture, presumably because blood breakdown products are pro-inflammatory and can also nourish bacteria [23]. Hammond et al. (2021) reported a CC rate of 18.2% in patients who experienced postoperative hematoma, compared to 8.8% in those who did not (*p* = 0.047) [5]. Likewise, a chronic seroma and/or mild surgical trauma can prolong the inflammatory phase of wound healing, increasing the likelihood of fibrosis.

The implant material itself contributes to the foreign-body response. Microscopic silicone leakage or shedding from older-generation implants was historically linked to high contracture rates, as free silicone droplets incite a foreign-body giant cell reaction in the capsule [31]. Modern cohesive gel implants minimize this microleakage (i.e., gel bleeding) phenomenon, and indeed studies show less silicone in contemporary capsules than in those from earlier implant generations, correlating with lower contracture incidence [31]. Mechanical factors also play a role: chronic implant micromotion and silicone particulate shedding can prolong the inflammatory phase and eventually lead to capsular fibrosis [32].

Implant surface texturing is another important modulator of the foreign-body response. In the breast augmentation setting, meta-analyses of randomized controlled trials have demonstrated that textured-surface implants significantly reduce capsular contracture rates compared to smooth implants (pooled OR 0.19; 95% CI, 0.07–0.52) [33], likely by disrupting the parallel alignment of myofibroblasts and collagen fibers within the capsule. Notably, this effect has not been replicated in the reconstruction setting, where concurrent ADM use likely confounds the comparison (see Section 5.1) [9].

### 3.3. Host Immune Response and Fibrotic Propensity

Recent research has focused on the specific immune pathways and host factors that determine why one patient’s foreign-body response remains mild while another’s becomes pathologic. Contracted capsules harbor increased pro-inflammatory (M1) macrophages and fewer anti-inflammatory (M2) macrophages compared to normal capsules, suggesting polarization towards a fibrogenic phenotype [32].

At the molecular level, IL-17 signaling and cellular senescence have been shown to synergistically drive the foreign body response; analysis of capsules from human breast implants revealed that IL-17-producing cells and senescent stromal cells sustain a feed-forward loop of chronic inflammation and fibrogenesis, and abrogation of IL-17 signaling significantly reduced fibrosis in preclinical models [34]. Similarly, fibrotic capsules show higher levels of Th17 cells (which secrete IL-17 and promote fibrosis) and reduced regulatory T-cells, indicating loss of normal immune regulation [35].

Gene expression analyses provide further support: a transcriptomic study by Mao et al. identified differential expression of dozens of genes in high-grade contracture capsules versus low-grade, many involving immune signaling and fibrosis pathways [36]. Notably, they found down-regulation of the *PRKAR2B* gene in severe contractures; this gene is linked to immune cell regulation, and its low expression was associated with significantly greater infiltration of M1 macrophages and follicular helper T cells in the capsule (r = 0.86 for each, *p* < 0.05) [36].

Histological analyses show that contracted capsules are typically thicker and contain more inflammatory cells (macrophages, lymphocytes) than non-contracted capsules [37]. In particular, high-grade (Baker III/IV—see below) capsules show dense, aligned collagen with contracted fibers and ongoing inflammatory infiltrate [37]. It is worth noting that a synovial-like inner lining has been described in some breast implant capsules; however, its prevalence, functional significance, and temporal evolution are inconsistent across studies, and its presence or absence is not reliably associated with contracture grade [37]. Accordingly, its presence should not be considered a defining histological feature of a healthy or pathological capsule. Thus, the concept of an inherent patient-driven propensity for fibrosis is gaining traction—and helps explain why only a subset of patients develop contracture even under similar conditions—though it remains an area of active research rather than a fully validated predictive mechanism. The bilateral presentation of CC in some patients further supports a patient-related etiology [7].

Clinically, the hypothesis that patients with a predisposition to hypertrophic scarring or keloid formation may be at higher risk for CC has been proposed, since such individuals tend to have elevated systemic pro-fibrotic cytokine levels [32]. However, hard evidence for a genetic predisposition predictive of CC remains limited and somewhat controversial—it is plausible but not yet clinically actionable.

### 3.4. Radiation-Induced Fibrosis

In post-mastectomy reconstruction patients, adjuvant radiotherapy is a well-established precipitating cause of CC. Radiation damages the microvasculature and cells of the chest wall tissues—a proven effect documented extensively in radiation biology literature—leading to hypoxia, cell death, and a profibrotic wound healing environment that affects the implant capsule [32]. Specifically, radiation injures the vascular endothelium, causing ischemia, decreased angiogenesis, and impaired collagen deposition that delays wound healing; myofibroblasts exposed to ionizing radiation lose integral negative feedback mechanisms, resulting in dysregulation and sustained fibrogenesis [5,32].

The clinical magnitude of radiation-associated CC risk—including quantitative estimates from large meta-analyses and the differential impact by reconstruction plane [38]—is detailed in Section 7; the present section focuses on the underlying biological mechanisms by which ionizing radiation promotes periprosthetic fibrosis. Histologically, irradiated capsules show exaggerated inflammation and fibrosis: a systematic review noted that capsules from radiated reconstructions were significantly thicker, more collagen-dense, and had more inflammatory cell infiltrate than those from non-radiated patients [37]. Radiation injury likely creates a profibrotic cytokine milieu (with elevated TGF-β, IL-6, etc.) and may impair the normal remodeling of the capsule, acting as an independent fibrotic stimulus that “primes” tissue for contracture [32].

## 4. Clinical Assessment and Classification

### 4.1. The Baker Classification

The primary method for evaluating CC remains clinical examination using the Baker classification, a four-grade scale originally introduced in 1978 [39]. Baker grades I through IV describe increasing firmness, implant palpability/visibility, and patient discomfort, with grade I being a soft, normal breast and grade IV indicating a hard, painful breast with obvious distortion. This simple grading system is deeply ingrained in clinical practice and is valued for its ease of use and familiarity [32]. It has been used both in clinical settings—to assess individual patients and guide surgical decision-making—and in research settings, where Baker scores are correlated with histological, immunological, and imaging data to study the pathophysiology and treatment of CC [21]. Spear and Baker’s 1995 modification subdivided grade I into “IA” (absolutely natural feel) and “IB” (palpable implant without visible firmness) to better reflect outcomes in reconstruction [40], as summarized in Table 1.

Despite its ubiquity, the Baker grading system is increasingly scrutinized for subjectivity and inconsistency. Recent evidence demonstrates poor interobserver reliability—de Bakker et al. (2020) found that the interobserver reliability of the Baker classification was poor (weighted κ = 0.55; 95% CI, 0.37–0.72), with exact agreement between two experienced plastic surgeons in only 48% of cases and discrepancies of more than one grade in 11% [21]. Remarkably, even when surgeons were asked to rate individual symptoms—firmness (κ = 0.64), dislocation (κ = 0.49), and symmetry (κ = 0.61)—separately, reliability remained poor, suggesting that the problem lies not merely in the composite grading but in the lack of consensus on what these symptoms entail and how to grade their severity [21]. Only four discrete categories are available, limiting granularity and making disagreement on mid-range grades (II vs. III) particularly common.

It is also worth noting that the original Baker scale was designed for cosmetic augmentation patients with intact breast tissue and does not fully account for the distinct tissue characteristics of the post-mastectomy setting, where native gland and subcutaneous fat have been removed. Furthermore, the Baker scale assesses the breast–implant complex as a whole, yet in prepectoral reconstructions with ADMs, a true periprosthetic capsule may not form; perceived “contracture” may instead reflect fibrosis of the overlying skin, fat, ADM, or muscle rather than a capsular process per se [41]. These limitations, combined with the finding that only approximately half of histological studies correlate their findings with Baker grades [37], highlight the need for a more standardized assessment framework. These collective limitations underscore that the Baker classification, while clinically pragmatic, introduces a systematic source of measurement imprecision that propagates through the evidence base: when the primary outcome measure is itself unreliable, even well-designed comparative studies may fail to detect true differences—or may report spurious ones. This measurement limitation should be considered a fundamental caveat when interpreting the comparative CC rates presented throughout this review.

### 4.2. The Role of Imaging

No imaging modality can independently diagnose CC; clinical examination according to the Baker scale remains the standard of care [32]. However, imaging serves as a valuable adjunct. Magnetic resonance imaging (MRI) can visualize peri-implant capsule thickness and detect pathological changes such as implant rupture, pericapsular fluid, and capsular calcification. Although the FDA historically recommended periodic MRI for silicone implant integrity screening, this guidance pertains primarily to (“silent”) rupture detection in the cosmetic setting and was not designed for CC assessment or the reconstructive population [42]. Importantly, the relationship between capsular thickness measured on MRI and clinical CC severity is inconsistent. Multiple regression analyses have failed to identify a statistically significant association between capsular thickness on MRI and Baker grade, although morphological features—such as increased implant roundness, solidity, and ratio length—do correlate with higher Baker grades (III/IV) and may hold promise as more sensitive indicators of contracture [43].

Ultrasound (US) is another commonly employed modality: high-resolution ultrasound can measure capsule thickness and identify pericapsular fibrosis, and it offers real-time, cost-effective assessment without radiation [32]. Kim et al. (2021) demonstrated that increasing capsular thickness on ultrasound was associated with higher Baker grades, with mean thickness increasing from ~0.6 mm in Baker grade II, to 1.07 mm in grade III and 1.89 mm in grade IV [41]. Thereby, ultrasound cut-off values for distinguishing between Baker grades I–II, II–III, and III–IV were proposed: 0.5 mm, 0.8 mm, and 1.2 mm, respectively [41]. Lastly and most recently, Oh et al. (2025) demonstrated that computed tomography (CT) can quantify CC by measuring changes in the ratio of implant projection to base diameter: for Baker grade III or IV, the projection-to-base ratio increased to ≥1.233, reflecting the spherical deformation of the implant under capsular constriction [44].

However, it is important to note that capsule thickness does not uniformly correlate with contracture—patients can have clinically significant contracture with a capsule of normal thickness, and conversely a thick capsule may remain soft. Therefore, imaging findings should be interpreted in context with the clinical examination rather than as standalone diagnostic criteria [32,43].

### 4.3. Emerging Objective Measurement Tools

There is broad recognition that an objective, reproducible scoring system for CC severity would greatly enhance diagnostic consistency. To date, no single objective metric has achieved universal acceptance. Sonoelastography—an advanced ultrasound technique that quantifies tissue stiffness—has emerged as one of the most promising candidate modalities. Both shear-wave and strain elastography can generate continuous stiffness values for the pericapsular tissues, with higher stiffness correlating with increasing Baker grade [43]. Shear-wave sonoelastography offers greater objectivity than strain elastography by using ultrasonic beams to generate transient vibrations, reducing operator dependence; early studies have found strong correlations between shear-wave scores and Baker grade (r = 0.89 in one series), although sample sizes remain small and prospective validation is lacking [43]. Notably, artificial intelligence–assisted interpretation of shear-wave elastography data has already demonstrated predictive value in other analogous clinical domains, such as prostate lesion characterization [45], suggesting that machine learning approaches could eventually enhance the diagnostic precision of elastography-based CC assessment.

Complementary physical measurement devices have also been investigated. Applanation tonometry—adapted from ophthalmology—measures breast firmness by quantifying the area of contact when a weighted disk is placed on the breast, offering an objective and inexpensive tool for longitudinal follow-up, although its specificity is limited when comparing different implant types [43]. Durometry determines the force required to deform the breast and has been shown to distinguish contracted from uncontracted breasts (Baker grade I = 0, II ≈ 0.2, III ≈ 2.0), though concurrent correlation with imaging measures has not been established [43]. Mammacompliance—a caliper-based technique that standardizes breast compression with known force—can differentiate Baker I from IV, with proposed cut-offs of <3.6 cm (no CC), 3.6–6 cm (mild CC), and >6 cm (severe CC), although these thresholds were not statistically validated [43].

A systematic review of all available objective measures concluded that no single metric captures the multifaceted nature of CC; instead, a multimodal approach combining clinical assessment, imaging, and quantitative measurement may be necessary to reliably characterize contracture severity [43]. The Baker grade, despite its limitations, is likely to remain the primary clinical tool for the foreseeable future, given its simplicity and widespread adoption. However, discordance between clinical grading systems and definitive histopathological findings is a recognized challenge across surgical disciplines; analogous discrepancies between biopsy and surgical specimen grading have been well-documented in oncologic contexts [46].

For research purposes—where unreliable measurement introduces substantial noise and hampers comparisons between studies—combining Baker grading with one or more objective modalities (e.g., US capsule thickness measurement, sonoelastography, or tonometry) and employing multiple blinded observers is strongly recommended [21,43]. The value of standardized imaging-based reporting frameworks for reducing interobserver variability has been well demonstrated: in bladder cancer staging, the introduction of the VI-RADS structured reporting system significantly improved diagnostic agreement among radiologists [47]. The development of an analogous consensus framework for CC assessment—incorporating clinical, imaging, and elastographic parameters—could similarly address the subjectivity that currently limits comparability across studies. Standardization of such a multimodal assessment framework remains an active and important area of investigation.

## 5. Implant-Related Factors and Surgical Adjuncts

### 5.1. Implant Surface: Smooth, Textured, and Polyurethane-Coated

Breast implant surfaces can be broadly classified into smooth, textured (micro- or macrotextured), nanotextured, and PU-coated. The International Organization for Standardization (ISO) defines surface roughness categories based on roughness average (Ra) values: smooth (<10 µm), microtextured (10–50 µm), and macrotextured (>50 µm). It should be noted that nanotextured implants are not currently designated as a formal ISO category; the term is used in the clinical and commercial literature to describe surfaces with Ra values in the nanometer range (approximately 4–6 µm or below), which are distinct from conventional microtextured surfaces in their tissue-interaction profile. Smooth implants have a polished shell with no intentional surface irregularities. Textured implants feature engineered surface roughness designed to promote tissue adherence and disrupt circumferential scar formation. PU-coated implants are covered with a layer of PU foam that gradually degrades in vivo, promoting a disorganized, less contractile collagen pattern in the capsule [48,49].

Textured implants were originally developed to reduce CC, as early studies in breast augmentation noted significantly lower contracture rates with textured surfaces compared to smooth (approximately 2–3-fold reduction in risk) [8]. However, in the specific context of post-mastectomy breast reconstruction, recent evidence indicates no significant difference in CC rates between smooth and textured implants [9]. A 2024 meta-analysis of IBBRs found CC odds nearly identical with smooth versus textured devices (OR 0.99, *p* = 0.97) [9]. This parity is likely confounded by the widespread concurrent use of ADM in modern reconstruction, which may itself modulate the fibrotic response and obscure any independent effect of surface texturing. Importantly, implant-associated anaplastic large-cell lymphoma (BIA-ALCL) has become a critical consideration in surface selection. This rare T-cell lymphoma has been overwhelmingly linked to textured implants [50]. Breast implant-associated squamous cell carcinoma (BIA-SCC) has also emerged as an additional rare malignancy associated with implant texturing [51]. Due to these risks, regulatory agencies have issued warnings or bans on certain macro-textured devices, leading to a shift toward smooth implants in many regions [50,52].

The Dutch Breast Implant Registry—DBIR (Harmeling et al. 2025) provides the largest population-based comparison of implant surfaces in reconstruction, including 3072 textured, 494 smooth, and 430 PU-covered implants [10]. At 4-year follow-up, cumulative incidence of revision for CC was 2.4% (95% CI 1.8–3.1) for textured, 4.7% (95% CI 1.8–9.8) for smooth, and 5.1% (95% CI 3.1–7.8) for PU implants (noting that only contractures requiring surgical revision were captured) [10]. In multivariable analysis, no significant differences were found between surface types for overall surface-related revision. However, subgroup analysis revealed that PU-covered implants had a significantly increased hazard of revision specifically for CC compared to textured implants (adjusted cause-specific hazard ratio [HRcs] = 2.49, 95% CI 1.24–5.01), as well as for asymmetry (HRcs = 2.31, 95% CI 1.33–4.02) [10], highlighting a potential signal that warrants further investigation. This finding contrasts with the historically favorable profile attributed to PU surfaces and may reflect several methodological considerations: the registry’s capture of only revision-requiring contractures (which may underrepresent clinically graded CC), differences in surgical practice across participating centers, the relatively low rate of prior radiotherapy (8.6%) in this cohort—which limits applicability to the oncologic population where PU implants are most commonly advocated—and the predominance of macrotextured implants as the comparator device in the Netherlands, introducing a surface-specific confounder.

Direct head-to-head comparisons of PU-coated implants against textured and ADM-covered devices are discussed in detail in Section 5.5. Table 2 summarizes the characteristics, CC profiles, and key considerations for each implant surface type.

### 5.2. Implant Filling: Silicone Versus Saline

Silicone gel-filled implants are by far the more commonly used in modern breast reconstruction owing to their natural feel and aesthetics [61]. Saline implants are very rarely used in Europe, where silicone dominates the market. Some reconstruction studies have suggested significantly lower CC rates with saline implants. In Christodoulou’s meta-analysis, patients who received saline implants had 81% lower odds of developing severe CC compared to those with silicone implants (OR 0.19, 95% CI 0.08–0.43) [9]; however, this estimate is based on only two reconstruction studies comprising 132 total cases, severely limiting statistical power and generalizability, and should be interpreted with caution [9]. Importantly, a companion meta-analysis by Haas et al. (2025) focusing on breast augmentation found no significant difference in CC between saline and silicone implants (OR 0.39, 95% CI 0.02–6.69, *p* = 0.52)—despite a much larger overall dataset [55]. This discordance between the augmentation and reconstruction settings may reflect differences in tissue coverage, surgical technique, or patient populations rather than a true implant-fill effect. Although the available evidence does not permit a definitive conclusion regarding a differential contracture risk between silicone and saline fill, any potential increase in contracture risk associated with silicone implants should be weighed against their well-established advantages in aesthetic outcomes—particularly relevant in the post-mastectomy setting where native breast tissue is absent and tactile quality of the reconstruction is highly dependent on implant properties.

### 5.3. Implant Shape: Round Versus Anatomical

Implant shape is another critical design element, classified mainly into round (spherical) versus anatomical (teardrop) implants. Anatomical implants are filled with a highly cohesive (“form-stable”) silicone gel and usually have a textured surface to prevent rotation [61]. Round implants can be either smooth or textured; if a round implant rotates, it does not alter the breast shape. Anatomical implants have been considered advantageous in women with minimal native breast tissue or thin mastectomy flaps, and may be particularly useful in unilateral reconstructions for matching the contralateral breast [61].

CC rates appear comparable between round and shaped implants when other variables are controlled. Khavanin et al. (2017) found no significant difference in CC, malposition, or overall reoperation rates between the two groups [62]. A comprehensive review by Han et al. (2020) noted that anatomical implants had a dramatically lower risk of visible rippling compared to round implants (relative risk 0.05) [58]. In current practice, smooth round implants remain the predominantly used devices for most IBBRs. While anatomical textured implants were extremely popular in the 2000s (comprising up to ~80% of implants in some European series [7]), many centers have transitioned to smooth round devices in light of BIA-ALCL concerns. The aesthetic differences can often be compensated for by surgical technique and adjunctive fat grafting [52,61].

### 5.4. Acellular Dermal Matrix: Role and Controversies

ADMs have transformed IBBR by enabling both expanded submuscular (dual-plane) and wholly prepectoral techniques. ADMs provide a biologic scaffold that modulates the foreign-body response and fibrosis. Available products include human cadaveric dermis (AlloDerm^®^, DermACELL^®^), porcine dermis (Strattice^®^), bovine pericardium (SurgiMend^®^), and others. A meta-analysis by Cook et al. (2024) reported that ADM use reduced the relative risk of CC by approximately 60–70% compared to reconstructions without ADM (risk ratio 0.3) [63]. However, the evidence is not uniformly supportive: the Swedish registry study by Hägglund et al. (2025) found no significant association between ADM use and contracture occurrence in prophylactic cases (adjusted HR 0.56, *p* = 0.54) [7], and a meta-analysis by Nolan et al. (2023) found that CC occurred in 6.8% of ADM cases versus 3.4% without ADM in prepectoral reconstructions—a non-significant difference [14]. Furthermore, a Swedish randomized controlled trial by Lohmander et al. (2021) found no significant difference in 2-year outcomes—including CC—between immediate reconstruction with partial muscle + ADM coverage versus total submuscular coverage without ADM, raising questions about the necessity of ADM in all reconstructive settings [15].

As noted above, the available ADM products differ in tissue of origin, processing method, and mechanical properties, yet no high-level evidence has established superiority of any single product in preventing CC [64]. Most ADMs are believed to act through a common mechanism of scaffold-mediated tissue remodeling, modulating macrophage polarization toward an anti-inflammatory (M2) phenotype and attenuating myofibroblast activation at the implant–tissue interface [32,63]. In practice, surgeons often base selection on availability, familiarity, and cost rather than on expected differences in capsule formation. Seroma remains the most common complication associated with ADM use, a factor that must be weighed against any potential anti-fibrotic benefit [14,15,16].

Synthetic and bioabsorbable meshes have been employed as cost-effective alternatives to biologic ADMs. These include fully absorbable meshes (e.g., Vicryl/polyglactin), partially resorbable meshes (e.g., TIGR^®^ matrix, which is resorbed over approximately 3 years while providing temporary mechanical support), and non-resorbable titanium-coated polypropylene meshes (e.g., TiLOOP^®^ Bra). Complication profiles differ across mesh types: absorbable meshes are associated with lower seroma rates than biologic ADMs, while non-resorbable meshes carry risks of mesh exposure, palpability, and chronic foreign-body reaction [65,66].

Regarding CC, Sobti et al. (2020) found no significant difference between AlloDerm and Vicryl mesh (OR 1.08, *p* = 0.91) [64]. A network meta-analysis by Murphy et al. (2023) similarly reported no significant differences in CC rates among biologic ADMs, synthetic meshes, and no-mesh approaches in prepectoral reconstruction [67]. TiLOOP^®^ Bra-assisted reconstruction has demonstrated acceptably low CC rates in single-center series, though randomized comparative data against biologic ADMs remain absent [66]. The choice between biologic and synthetic scaffolds thus currently rests on cost, availability, seroma profile, and surgeon experience rather than a demonstrated difference in contracture prevention. Given the limitations and costs associated with ADM, an important alternative approach—the use of PU-coated implants without ADM coverage—has attracted increasing attention and is discussed in the following section.

### 5.5. Polyurethane-Coated Implants Versus ADM-Covered Implants

A growing body of retrospective single-center data has compared PU-coated implants (used without ADM) to ADM-covered or textured implants in prepectoral reconstruction. While these data generate hypothesis-forming comparisons with potential cost implications, it is important to note that the evidence base consists entirely of retrospective single-institution series (Level of Evidence 4), and conclusions should be interpreted accordingly. Salgarello et al. (2025) compared 70 PU breasts to 65 ADM-covered breasts in immediate prepectoral reconstruction [54]. PU implants were associated with significantly fewer total complications (25.7% vs. 63.1%, *p* < 0.001), lower seroma rates (2.9% vs. 33.8%), and lower infection rates (1.4% vs. 6.2%). At 5 years, severe CC (Baker III–IV) was markedly higher in the ADM group, particularly in non-irradiated patients (48.4% vs. 4.2% for Baker III–IV, *p* < 0.001) [54]. Aesthetic outcomes were also superior in the PU group, though the ADM group showed advantages in rippling and implant visibility [54]. Among irradiated patients, both groups showed high rates of progressive contracture, though numbers were small. These findings suggest that PU-coated implants may offer a safer and more cost-effective approach for prepectoral reconstruction, though prospective multi-center trials are needed to confirm these results.

Earlier comparative data from Loreti et al. (2020) also support a favorable profile for PU implants, albeit against textured rather than ADM-covered implants. In their series of 358 DTI reconstructions, PU implants achieved a Baker III–IV contracture rate of 8.1% compared to 15.8% with textured implants (*p* = 0.009) over a median 2.3-year follow-up [53]. The benefit was particularly pronounced in irradiated patients, where PU implants showed a hazard ratio of 0.3 relative to textured implants for severe contracture (*p* = 0.003) [53]. The single-center retrospective data from Loreti et al. [53] and Salgarello et al. [54] suggest a potentially favorable CC profile for PU-coated implants in non-irradiated prepectoral reconstruction, though effect sizes may be inflated by institutional case selection, short or variable follow-up, and non-standardized outcome definitions. Importantly, the largest available dataset—the DBIR (Harmeling et al., *n* = 3996)—found a significantly higher cause-specific hazard for CC-requiring revision with PU implants compared to textured devices (HRcs = 2.49) [10], a finding that tempers enthusiasm and highlights the need for prospective multicenter trials, with standardized assessment criteria (Table 3), before PU implants can be endorsed as superior to ADM-covered reconstructions [10,53,54].

## 6. Surgical Technique and Modifiable Factors

Before examining individual surgical and patient-related variables, Table 4 consolidates the key findings from major meta-analyses and registry studies on modifiable risk factors for CC, providing an evidence-based overview of the comparative data discussed throughout this and subsequent chapters.

### 6.1. Mastectomy Incision Type

The surgical incision used for mastectomy (particularly nipple-sparing mastectomy) can significantly influence flap perfusion and complication rates, which in turn may affect CC development. Common approaches include the inframammary fold (IMF) incision, lateral or radial incisions, and periareolar incisions. Periareolar incisions carry the highest complication rates and are theoretically linked to higher contracture risk through greater bacterial introduction from breast ducts [30]. In the reconstruction setting specifically, Hammond et al. (2021) reported no significant difference in CC rates when comparing simple mastectomy, skin-sparing mastectomy, and nipple-sparing mastectomy techniques (*p* = 0.234) [5]. This contrasts with the augmentation literature, where periareolar approaches are associated with higher contracture risk, and may reflect the fact that in mastectomy the majority of ductal tissue is removed regardless of incision approach, reducing the relevance of incision-related bacterial introduction. Nonetheless, the principle of minimizing contamination through avoidance of ductal tissue transection remains sound when applicable. Although no large trials have specifically stratified CC rates by mastectomy incision type in reconstruction, the principle of minimizing bacterial contamination favors approaches that avoid transecting ductal tissue.

Regardless of incision approach, perioperative infection control measures targeting the biofilm hypothesis are an important component of CC prevention. In practice, meticulous skin prepping, nipple shields, plastic adhesive drapes, implant pocket irrigation with antibiotic and/or antiseptic solutions, and minimal implant handling are routine [28,29]. Clinical data support some of these measures—for instance, periareolar incisions (prone to contact with breast duct flora) have higher rates of contracture than inframammary incisions, presumably due to greater bacterial introduction [30]. A meta-analysis reported a pooled relative risk of 0.47 (95% CI 0.32–0.71) for CC with intraoperative antibiotic irrigation compared to without [5].

Emerging registry data further support the role of perioperative infection control: in the Swedish national registry study by Hägglund et al. (2025), postoperative prophylactic antibiotic treatment was associated with a significantly reduced risk of CC in univariable analysis (HR 0.18, 95% CI 0.04–0.89, *p* = 0.035), although this association did not retain statistical significance in the multivariable model after adjustment for device type and reconstruction timing [7].

### 6.2. Implant Placement Plane: Prepectoral Versus Subpectoral

The choice between prepectoral and subpectoral implant placement is one of the most actively debated variables in IBBR, with implications for CC, animation deformity, soft-tissue aesthetics, and patient-reported outcomes. The evidence base has evolved substantially over successive meta-analyses, and the contemporary consensus points toward equivalent CC rates between planes (in the current ADM era), with meaningful trade-offs in other complications.

Early pooled analyses suggested a prepectoral advantage for CC. In a 2020 meta-analysis of 15 studies comprising 1868 patients, Li et al. reported a significantly lower CC rate in the prepectoral group (OR 0.45, 95% CI 0.27–0.73, I^2^ = 0%), though overall complication rates and implant loss were comparable between planes [68]. This initial finding was corroborated by Ostapenko et al. (2023), who pooled 10 studies reporting CC data from 15 comparative studies (3101 patients) and likewise found significantly lower CC with prepectoral placement (OR 0.54, 95% CI 0.32–0.92, *p* = 0.02, I^2^ = 53%), along with markedly lower animation deformity (OR 0.02, 95% CI 0.00–0.25) and prosthesis failure (OR 0.61, 95% CI 0.44–0.84) [11]. However, these early estimates were derived from studies with a mean follow-up of only 19 months and without stratification by ADM use. This is a critical confounder in the studies included in these early meta-analyses: within the reported cohorts, prepectoral cases almost invariably employed ADM while subpectoral cases frequently did not, meaning the apparent plane-related advantage may partly reflect the anti-fibrotic properties of ADM rather than implant plane per se. It is acknowledged that prepectoral reconstruction without ADM is practiced at some centers, and ADM utilization patterns vary substantially across institutions and countries.

Subsequent larger analyses have consistently failed to replicate these early findings. The Christodoulou et al. (2024) meta-analysis, which focused specifically on CC as the primary outcome, pooled 16 studies (3499 cases) comparing subpectoral and prepectoral placement and found no significant difference (OR 1.21, 95% CI 0.75–1.95, *p* = 0.44), with low heterogeneity (I^2^ = 26%) [9]. Importantly, subgroup analyses stratified by ADM use also showed no significant difference in any stratum, suggesting that the earlier prepectoral advantage may have been confounded by differential ADM utilization across planes [9]. Similarly, Zhu et al. (2023) compared prepectoral to partial subpectoral ADM-assisted reconstructions across 10 studies (2667 reconstructions) and found no significant difference in CC (RR 0.939, 95% CI 0.678–1.300, *p* = 0.703), with pooled incidence rates of 5.8% and 4.7% for prepectoral and partial subpectoral groups, respectively [13]. Subsequently, Wu et al. (2024), in the largest meta-analysis at the time of its publication (40 studies, 12,943 breasts), confirmed no significant difference in CC between planes [12]. Most recently, Pumilia et al. (2025) pooled 47 studies encompassing 8350 patients and 12,074 breasts and likewise found no significant difference in CC between prepectoral and submuscular groups [69]. The convergence of evidence from these six successive meta-analyses indicates that CC rates are at worst equivalent between planes in the contemporary reconstructive setting, and no modern pooled analysis shows that prepectoral placement increases contracture risk.

At the single-institution level, Sobti et al. (2020) reported lower CC rates with prepectoral placement in an exclusively irradiated DTI cohort (30.0% vs. 51.8%, adjusted OR 0.24, 95% CI 0.08–0.64, *p* < 0.01), with no independent effect of ADM type on CC [64]. While the small sample size (*n* = 81 breasts) and retrospective design limit generalizability, this study contributed an important mechanistic observation: in subpectoral patients with radiation-associated contracture, administration of a muscle relaxant under general anesthesia produced descent of the contracted breast mound to a symmetric position, implicating pectoralis major muscle fibrosis—rather than capsule alone—as a major pathogenic contributor to CC in the subpectoral irradiated setting [64]. This physiological observation supports the biological rationale for prepectoral placement in patients anticipated to receive PMRT, though it requires confirmation in larger prospective series.

While CC rates appear equivalent between planes, the choice of implant placement involves genuine trade-offs in other complications. Prepectoral reconstruction was associated with significantly higher rates of seroma (OR 1.41, 95% CI 1.08–1.85) and visible rippling (OR 2.21, 95% CI 1.52–3.21) in the latest Pumilia meta-analysis [69], findings corroborated by Wu et al. (seroma OR 1.55, 95% CI 1.02–2.35; rippling OR 2.39, 95% CI 1.53–3.72) [12]. The higher seroma rate with prepectoral placement may relate to the larger volume of ADM typically used for complete implant wrapping in prepectoral cases compared with the inferior sling employed in dual-plane reconstructions, though the true etiology remains undetermined [69].

Rippling reflects the thinner soft-tissue coverage anteriorly when the pectoralis muscle is not interposed, particularly in patients with thin mastectomy skin flaps, though this complication is often amenable to correction with autologous fat grafting [69]. The Nolan et al. (2024) systematic review, focused specifically on reconstruction after nipple-sparing mastectomy, reported descriptive CC rates of 4.8% for total submuscular, 0.3% for dual-plane, and 3.1% for prepectoral reconstruction, alongside a rippling rate of 10.6% in the prepectoral cohort versus 0.9–4.8% in submuscular planes [71]. Rates of hematoma, infection, wound healing complications, mastectomy flap necrosis, nipple necrosis, implant exposure, explantation, and readmission were not significantly different between planes across the major meta-analyses [12,13,69].

Aside from contracture, animation deformity—distortion of the reconstructed breast with pectoral muscle contraction—is the most consistent differentiator between the two planes. Subpectoral implants can adhere to the overlying muscle, causing visible motion deformities that 20–80% of patients report as bothersome in surveys. Prepectoral reconstruction essentially eliminates this problem. Ostapenko et al. reported an extraordinarily low pooled OR of 0.02 (95% CI 0.00–0.25, *p* = 0.002) for animation deformity with prepectoral placement, though with substantial heterogeneity (I^2^ = 73%) [11]. Zhu et al. reported pooled animation deformity incidences of 1.2% in prepectoral versus 29.7% in partial subpectoral ADM reconstructions (RR 0.040, 95% CI 0.002–0.853, *p* = 0.039) [13]. Wu et al. found a significantly reduced incidence with prepectoral placement (OR 0.37, 95% CI 0.19–0.70, *p* = 0.003) [12], and Pumilia et al. confirmed this with an OR of 0.09 (95% CI 0.03–0.25) [69]. This finding has been replicated across all available pooled analyses, making the virtual elimination of animation deformity the principal functional advantage of the prepectoral approach.

Patient-reported outcomes provide additional nuance. In the Nolan review (2024), BREAST-Q satisfaction scores ranged from 60 to 93 across all domains for all planes, with physical well-being of the chest numerically highest in the prepectoral cohort (78.3 vs. 66.8–69.1 in submuscular planes) [71]. Zhang et al. (2025), in a comparative study of 136 breast reconstructions, found significantly better BREAST-Q scores for physical well-being (chest function preservation) and psychological well-being in the prepectoral group, while satisfaction with breast appearance and sexual well-being were similar between planes [72]. These data suggest that the reduced pain and muscle impairment associated with prepectoral placement translates into measurable quality-of-life benefits, even when aggregate satisfaction scores are broadly comparable.

It is important to distinguish the reconstruction setting from cosmetic breast augmentation, where the tissue biology differs fundamentally. In the augmentation setting, where the mammary gland remains intact, subpectoral (submuscular) placement is associated with significantly lower CC rates than prepectoral (subglandular) placement. In the augmentation setting, the meta-analysis by Haas et al. (2025; 24 studies, 17,707 cases) reported an OR of 0.35 (95% CI 0.25–0.50, *p* < 0.00001) favoring subpectoral placement [55]. Similarly, Li et al. (2019) found significantly higher CC rates in the prepectoral (subglandular) augmentation group [73]. However, this comparison is not directly applicable to post-mastectomy reconstruction, where no glandular tissue remains after mastectomy. The presence of non-sterile breast ductal tissue in the subglandular plane—absent after mastectomy—may partly explain the higher augmentation contracture rates through enhanced biofilm formation and chronic subclinical inflammation. In the post-mastectomy setting, the relevant comparison is between subpectoral and prepectoral (with or without ADM), and here the convergence of meta-analytic evidence indicates equivalent CC outcomes [9,11,12,13,69].

In summary, the current evidence indicates that prepectoral and subpectoral implant placement carry similar CC risks in the reconstruction setting, particularly in the contemporary ADM era. The choice between planes involves a trade-off profile: prepectoral placement virtually eliminates animation deformity and confers advantages in chest wall comfort and physical well-being, but at the cost of higher rates of seroma and visible rippling, particularly in patients with thin skin flaps. These data support an individualized approach to plane selection based on patient anatomy (skin flap thickness), anticipated adjuvant therapy, and informed patient preference regarding the differing complication profiles of each approach.

### 6.3. Direct-to-Implant Versus Two-Stage Reconstruction

In IBBR, two surgical pathways are commonly used: a DTI approach and a traditional two-stage approach (temporary TE followed by exchange to a permanent implant). A recent systematic review and meta-analysis including over 21,500 patients found no significant differences in total complication rates, patient satisfaction, or aesthetic outcomes between one-stage and two-stage implant reconstructions in appropriately selected patients [19]. CC rates are comparably low in the absence of adjuvant radiation. In the Swedish registry study by Hägglund et al. (2025), which enrolled women undergoing risk-reducing (prophylactic) bilateral mastectomy with immediate implant-based reconstruction—predominantly using fixed-volume silicone implants with or without ADM, via both prepectoral and subpectoral approaches—severe contracture (Baker III–IV requiring surgery) was observed in approximately 3.6% of reconstructions at 1 year, rising to 4.7% at 5 years, with no statistically significant difference between one-stage DTI and two-stage TE-based timing approaches [7]. Importantly, they observed that reconstructions involving a permanent TE (combined expander-implant device) had dramatically higher odds of severe CC compared to fixed-volume implants (adjusted HR 19.3, 95% CI 3.9–95.4, *p* < 0.001), underscoring the substantial impact of device type on CC incidence and highlighting that device selection may outweigh reconstruction timing as a determinant of contracture risk in some clinical contexts [7].

### 6.4. Patient-Related Risk Factors

Beyond surgical technique and implant characteristics, patient-level factors may contribute to CC risk. In the largest database analysis to date, Ali et al. (2023) examined 6547 patients undergoing alloplastic reconstructive and augmentation mammaplasty from the National Surgical Quality Improvement Program (NSQIP) and identified both older age (OR 1.10 per year, 95% CI 1.09–1.10, *p* < 0.001) and higher body mass index (OR 1.12, 95% CI 1.10–1.13, *p* < 0.001) as independently associated with CC requiring capsulectomy [74]. Cancer diagnosis was the strongest comorbidity predictor in their model (OR 7.71, 95% CI 2.22–26.8, *p* = 0.001), likely reflecting the confounding influence of radiotherapy and more extensive surgical procedures in oncologic patients [74].

However, these findings require careful contextualization. Hammond et al. (2021), in a cohort exclusively comprising reconstruction patients, found that age was not a significant risk factor for CC, suggesting that the age association observed in combined augmentation/reconstruction databases may be confounded by operative indication—older patients are more likely to have cancer necessitating radiation, rather than age itself promoting fibrosis [5]. Similarly, neither BMI nor active smoking reached statistical significance in the Hammond reconstruction cohort [5], and the Ali NSQIP analysis likewise found no association with current smoking (OR 1.04, 95% CI 0.86–1.26, *p* = 0.681) [74]. This contrasts with cosmetic augmentation literature, where some studies have implicated smoking as a risk factor, and likely reflects differences in tissue biology, surgical technique, and follow-up between the two populations.

Wound infection was the most common postoperative complication associated with capsulectomy in the NSQIP cohort (OR 6.69, 95% CI 1.74–25.8, *p* = 0.006) [74], reinforcing the connection between infectious complications and capsular fibrosis discussed in Section 3.1. The role of postoperative hematoma as a precipitating factor for contracture is further supported by Hammond et al., who observed a CC rate of 18.2% in patients with hematoma compared to 8.8% in those without (*p* = 0.047), although this did not retain significance on multivariate analysis [5]. These data collectively suggest that while patient demographics play a modulating role, the dominant risk factors for CC in reconstruction remain procedure-related (radiation, device type) rather than patient-intrinsic.

## 7. Radiotherapy and Capsular Contracture

### 7.1. The Impact of Postmastectomy Radiotherapy

PMRT is a crucial adjunct in breast cancer treatment but is well documented to exacerbate fibrosis and CC in IBBR [38]. An updated 2025 meta-analysis by Ogita et al. reported that PMRT nearly ten-fold increased the odds of CC (pooled OR ≈ 9.6, 95% CI 5.8–16.1) [18]. Awadeen et al. found a risk ratio of 5.17 (*p* = 0.001) for CC in prepectoral reconstructions receiving PMRT versus those that did not [75]. Parmeshwar et al. (2025) reported the odds of CC were approximately 8.9-fold higher with radiation in DTI prepectoral reconstructions (*p* < 0.001) [70]. In practical terms, reported rates of severe contracture in irradiated implant reconstructions range from ~20–50%, far higher than in non-irradiated cases (often <10%) [17,18]. Cosmetic outcome scores are significantly poorer in irradiated reconstructions: the pooled OR for poor cosmesis was 3.55 (95% CI 1.80–6.98) based on six studies comparing PMRT to no PMRT [18]. These findings, together with the broader evidence based on modifiable risk factors, are consolidated in Table 4.

### 7.2. Timing of Radiation: Expander Versus Implant Irradiation

A central dilemma in irradiated reconstruction is whether to deliver PMRT to a TE (with delayed implant exchange) or to a permanent implant placed immediately. Cordeiro et al. reported that patients who received PMRT to a permanent implant had severe contracture in 50.9% of cases, versus only 17.1% in those irradiated at the expander stage [20]. Ma et al. (2024) similarly found a significantly higher rate of severe contracture in a DTI-PMRT group (37.4%) compared to a TE-PMRT group (24.0%; *p* = 0.039), though reconstruction failure was lower in the DTI group (9.1% vs. 19.2%) [76]. The 2025 meta-analysis by Ogita et al. quantified this dichotomy: PMRT to an expander was associated with significantly lower odds of severe CC (OR = 0.33, 95% CI 0.12–0.92) but higher odds of reconstruction failure (OR = 2.33, 95% CI 1.43–3.82) [17]. No difference in overall major complication rates was observed between the two timing strategies [17].

The reduced contracture in two-stage reconstruction is partially attributed to surgical factors—at the time of expander-to-implant exchange, surgeons often perform capsulectomy/capsulotomy, thereby physically reducing fibrosis [38]. Iwahira et al. (2025), in a single-surgeon cohort of 340 irradiated patients, found that radiation timing was not a determinant of CC development; instead, post-irradiation skin redness, implant mobility, skin pinchability at one year, and expander positioning were the significant predictive factors [77]. This underscores that the local tissue response to radiation—rather than merely the timing—may drive contracture formation.

Given these trade-offs—lower contracture but higher reconstruction failure with expander irradiation, and vice versa—there remains controversy and no universal consensus on optimal sequencing [78]. Patient-specific priorities (avoiding multiple surgeries versus maximizing cosmetic outcome) must be weighed. High-level evidence (randomized trials) is lacking, but current data underscore that radiation timing is a critical determinant of CC incidence [17,76].

### 7.3. Radiotherapy Fractionation and Advanced Techniques

In recent years, moderately hypofractionated PMRT (e.g., 40–42.5 Gy in 15–16 fractions) has been increasingly adopted in lieu of conventional fractionation (50 Gy in 25 fractions). Emerging evidence suggests hypofractionation is equally safe in the context of implant reconstructions. Michaeli et al. reported major implant complication rates of 24.1% with hypofractionated PMRT versus 33.3% with conventional fractionation (not significant) [79]. These findings are consistent with early results from randomized trials such as FABREC, which reported no significant increase in reconstruction complications or CC rates with hypofractionated PMRT compared to standard fractionation [80]. International expert panels now largely endorse hypofractionated PMRT even for patients with immediate reconstruction—in a recent DEGRO survey, >85% of experts voted that moderate hypofractionation should be offered regardless of reconstruction status [38].

Advanced delivery techniques, such as intensity-modulated radiation therapy (IMRT), volumetric-modulated arc therapy (VMAT), and proton beam therapy, offer improved dose conformality and reduced hotspots, which could theoretically lower the propensity for focal fibrosis [81]. Early clinical experience with proton PMRT is promising—Gao et al. (2025) reported low reconstructive failure rates (~7.6%) and no increase in surgical complications with proton therapy [82]. However, direct comparative data on CC between proton and photon PMRT remain limited. At present, the choice of radiation modality is often driven by resource availability and the goal of sparing organs at risk rather than specific evidence of reducing contracture.

### 7.4. Oncologic Therapy Interactions

While PMRT is the most impactful oncologic factor, emerging evidence suggests that systemic cancer therapies may also modulate CC risk. Hammond et al. (2021), in a multivariable analysis of 451 reconstruction patients, reported that adjuvant chemotherapy was independently protective against CC (OR 0.289, 95% CI 0.114–0.731, *p* = 0.01) [5]. This is a notable finding from a single institution that, to our knowledge, has not yet been independently replicated. The proposed mechanism involves the anti-fibrotic effects of taxane-based regimens: exposure of fibroblasts to paclitaxel and docetaxel significantly impairs fibroblast migration by disrupting intracellular microtubule dynamics, potentially attenuating the excessive collagen deposition that characterizes CC [5]. If substantiated in prospective studies, this observation could have implications for risk stratification—patients receiving adjuvant chemotherapy may have a somewhat lower baseline contracture risk, potentially influencing reconstructive planning.

Neoadjuvant chemotherapy was associated with higher CC rates in univariate analysis in the same cohort (*p* = 0.006), but this association was not supported on multivariate analysis and was attributed to confounding by selection: patients receiving neoadjuvant chemotherapy had a significantly higher utilization of subsequent PMRT (*p* < 0.001) [5]. This underscores the importance of controlling for radiation status when evaluating the effects of systemic therapies on capsular outcomes.

The role of adjuvant hormonal therapy remains less clear. Hammond et al. found no significant association between selective estrogen receptor modulators (tamoxifen) or aromatase inhibitors and CC rates [5]. However, preclinical and translational data suggest a potential anti-fibrotic role for tamoxifen: Li et al. (2025) reported that among patients undergoing endocrine therapy, tamoxifen was least associated with severe contracture (27.8%) and was most significantly negatively correlated with contracture severity (*p* < 0.0001), and in animal models, tamoxifen reduced capsular thickness by 59% compared to controls, likely through inhibition of myofibroblast TGF-β1 production [83]. These data remain preliminary and require validation in dedicated clinical trials, but they raise the intriguing possibility that concurrent oncologic pharmacotherapy may inadvertently attenuate periprosthetic fibrosis.

## 8. Clinical Outcomes and Impact of Capsular Contracture

It is well-established that CC adversely affects cosmetic results. Patients who develop CC will have significantly worse breast aesthetics compared to those who do not. Clinical series confirm this: in a Danish cohort, patients with postoperative complications reported clearly lower cosmetic satisfaction, an effect more pronounced in IBBRs than autologous flaps [84]. CC also diminishes patient satisfaction and quality of life. A 2023 patient-reported outcomes study found that patients with CC had significantly lower BREAST-Q scores across multiple domains, including psychosocial, sexual, and breast satisfaction (*p* = 0.015) [85].

CC frequently necessitates reoperation. Baker grade III/IV contractures typically require surgical intervention (capsulotomy or capsulectomy) to relieve the contracture and improve breast shape [86]. CC has been identified as one of the leading causes of late revision surgery, second only to infection in many reports [59,86]. Reconstructions complicated by CC have a higher risk of ultimate implant loss; a meta-analysis of 5216 implant reconstructions found that cases with CC contributed to nearly two-fold higher odds of implant loss [87].

The temporal trajectory of CC development is an important clinical consideration. The Swedish registry study by Hägglund et al. (2025) demonstrated that the majority of contractures (82%) occurred within the first two years after reconstruction, with cumulative incidence rising from 1.9% at 1 year to 4.7% at 5 years, after which it remained stable through 8 years of follow-up [7]. These registry data from prophylactic reconstruction patients suggest a plateau in contracture risk after 5 years in non-irradiated patients. However, other studies have observed continued CC development over longer periods, with Meshkin et al. (2023) noting an estimated additional 1% risk per year and a cumulative 24.6% incidence over 10 years in a broader breast implant population encompassing both augmentation and reconstruction [88]. These differing temporal profiles likely reflect differences in patient populations, radiation exposure, and implant types, and underscore the need for long-term follow-up protocols.

Regarding long-term trajectory, emerging data have challenged the traditional assumption that implant-based results invariably deteriorate over time. In a 12-year follow-up study of over 2200 two-stage reconstructions, aesthetic outcomes and patient-reported well-being remained remarkably stable over time [89]. Surgeon-rated aesthetic scores stayed in the “very good” range even at 10+ years, and patient satisfaction did not decline with longer follow-up—in fact, some metrics improved at 5–10 years post-op [89]. These findings challenge the traditional surgical dogma that IBBR results inevitably deteriorate as capsular fibrosis progresses. While some capsular change is expected, the data suggest that with timely capsular interventions (capsulotomy, fat grafting) and appropriate maintenance, patients can achieve durable satisfaction.

## 9. Management of Established Capsular Contracture

When clinically significant CC develops (Baker grade III–IV), management is predominantly surgical. The current standard approach involves some combination of capsulectomy or capsulotomy, implant exchange, and—when feasible—a change in implant plane or pocket [4,59]. However, the optimal surgical strategy remains debated, and non-operative pharmacological approaches are emerging as adjunctive therapies for early-stage contracture.

### 9.1. Surgical Management

Capsulectomy (partial or total removal of the fibrotic capsule) has traditionally been considered the gold standard for surgical treatment of CC [4]. Total capsulectomy, however, is more technically demanding and carries higher morbidity, including risk of bleeding and pneumothorax, compared to capsulotomy (incision and release of the contracted capsule) [59]. Recurrence rates after both techniques are variable and broadly comparable, ranging from 0 to 54% in the literature [32,59]. In a systematic review, Wan and Rohrich found inadequate evidence to support the superiority of capsulectomy over open capsulotomy, or of total over partial capsulectomy, concluding that surgical choice should be tailored to the individual patient and clinical presentation [90].

Implant exchange is a critical component of revisionary surgery: Wan and Rohrich reported that implant exchange was associated with lower recurrence rates (0–26%) compared to procedures without exchange (0–54%), likely because replacing the implant eliminates the biofilm-colonized device [59]. Change in the implant plane (e.g., from subpectoral to prepectoral, or vice versa) has been associated with reduced recurrence rates when combined with capsulectomy [32,59]. For patients with a premuscular implant, capsulectomy with site change to a submuscular pocket is generally favored; for those with submuscular placement, partial capsulectomy or capsulotomy may suffice to minimize surgical morbidity [4].

The use of ADM at revision for CC has emerged as a promising adjunctive therapy. Hidalgo and Weinstein demonstrated that the incorporation of ADM in revision capsulectomy successfully treated CC in over 85% of cases, presumably by disrupting the circumferential fibrotic cascade and modulating the local immune response at the implant–tissue interface [91]. However, prospective data comparing revision outcomes with and without ADM are limited, and the added cost and complication profile (particularly seroma) must be weighed against the potential benefit. A detailed comparison of surgical techniques for CC management (including variations in capsulectomy extent, neopocket formation, and conversion strategies) is beyond the scope of this pathophysiology- and prevention-focused review; the reader is referred to dedicated surgical management reviews by Wan and Rohrich [90] and Gorgy et al. [59] for comprehensive technical guidance.

### 9.2. Autologous Fat Grafting

Fat grafting has been explored both as a preventative measure against CC development and as a therapeutic adjunct to surgical revision or standalone intervention for mild established contracture. In the preventative context, prophylactic lipofilling of the mastectomy flap at the time of or subsequent to reconstruction may improve flap vascularization, attenuate the local inflammatory environment, and reduce the fibrogenic stimulus at the implant–tissue interface—effects proposed to be mediated by paracrine activity of transplanted adipose-derived stem cells [92]. However, prospective evidence for a preventative effect remains limited. Papadopoulos et al. (2018) demonstrated that lipofilling can reduce CC grade and alleviate fibrotic damage, particularly in radiation-induced contracture [92]. The proposed mechanism involves the paracrine effects of adipose-derived stem cells, which secrete anti-inflammatory and antifibrotic cytokines that modulate the local tissue environment [59]. While these findings are promising, current evidence is based on small series with high risk of bias, and larger controlled studies are needed to establish the role of fat grafting in the management algorithm for CC.

### 9.3. Pharmacological Approaches

Non-operative pharmacological strategies targeting the inflammatory and fibrotic cascades underlying CC have attracted increasing interest, although most remain investigational or are used off-label. Leukotriene receptor antagonists (Montelukast and Zafirlukast), typically used in asthma management, have been the most extensively studied pharmacological agents for CC. These agents inhibit cysteinyl leukotrienes C4, D4, and E4, which are potent inflammatory mediators implicated in periprosthetic fibrosis [59]. A meta-analysis by Wang et al. (2020) demonstrated that leukotriene antagonists have significant effects in both preventing and treating CC [93]. These medications have been reported to be efficacious in improving and resolving contracture symptoms when employed during early stages of symptom development (Baker II–III) [88]. However, hepatotoxicity is a potential concern, necessitating monitoring of transaminase levels, and long-term efficacy and safety data remain limited [59].

## 10. Future Directions

Several emerging insights and research directions aim to further improve outcomes related to CC in IBBR. One area of innovation is implant surface technology. Nanotextured expanders and implants have shown encouraging results—a 2024 study found that nanotextured TEs were associated with dramatically lower odds of CC (OR 0.12, *p* < 0.001) compared to conventionally textured devices, even in the setting of radiation [60]. The comparison of PU-coated implants versus ADM-covered implants is another priority area, with the potential to reduce both complications and costs [54].

Beyond nanotexturing, a broader range of surface modification strategies is under active preclinical investigation. Li et al. (2025) comprehensively reviewed emerging approaches, including carbon ion implantation combined with asiaticoside (which inhibited fibroblast activity and capsular formation), polydopamine grafting with collagen to enhance mesenchymal stem cell adhesion, 2-methacryloyloxyethyl phosphorylcholine (MPC)-functionalized polydimethylsiloxane (PDMS) coatings that reduce macrophage activation, and polycarboxybetaine coatings that attenuate and modulate the foreign-body response [83]. While none of these technologies has progressed to clinical trials in the breast reconstruction setting, they represent a rich pipeline of innovation aimed at fundamentally altering the implant–tissue interface to reduce and modulate the dysregulated fibrotic cascade from the point of first contact, without seeking to eliminate the physiologically necessary foreign-body response itself.

Conversely, several pharmacological agents investigated for CC prevention or treatment remain at early or investigational stages and are appropriately considered future directions rather than established clinical tools. These include:

1. Pirfenidone—this antifibrotic drug, approved by the FDA for idiopathic pulmonary fibrosis, has shown promise in both preclinical and early clinical studies. Gancedo et al. demonstrated prevention of CC in animal models by modulating TGF-β levels [94], and a subsequent controlled clinical trial by Veras-Castillo et al. showed reduction in contracture in all enrolled patients (*n* = 17), with effects persisting after medication discontinuation [95].
2. Botulinum toxin A (BTX-A)—has been investigated for its potential to inhibit CC through multiple mechanisms: it blocks the TGF-β1/Smad signaling pathway, reduces type I and type III collagen deposition, and inhibits differentiation of fibroblasts into myofibroblasts [96]. A systematic review by Li et al. (2018) evaluated the use of BTX-A in implant-based breast surgery and concluded that it may control postoperative pain, accelerate tissue expansion, and relieve contracture deformities [96]. However, the optimal dose, injection timing, and frequency remain unquantified, and most evidence derives from small non-randomized studies. Larger randomized controlled trials and mechanistic studies are needed before BTX-A can be recommended for routine clinical use in CC prevention [96].
3. Other agents—additional pharmacological strategies under investigation include COX-2 inhibitors (diclofenac dermal patches have shown benefit in Baker II–III contracture) [88], cromolyn sodium (a mast cell stabilizer that reduced inflammation and capsular thickness in animal models) [59], and statins (simvastatin has demonstrated reduction in capsular fibrosis around silicone implants in preclinical studies) [96]. While these agents offer biologically plausible mechanisms of action, none has progressed to the stage of high-quality clinical evidence sufficient to support routine use. Future clinical trials should evaluate these pharmacological approaches alongside standardized surgical management to determine their role in a multimodal prevention and treatment strategy. A comprehensive appraisal of the pharmacological evidence for each agent—including dosing protocols, safety profiles, and regulatory status—is beyond the scope of this review; the reader is referred to dedicated reviews by Gorgy et al. [59] and Meshkin et al. [88] for detailed pharmacological guidance.

Objective assessment tools—including shear-wave elastography, applanation tonometry, and standardized ultrasound protocols—are being investigated for early detection of capsular thickening or stiffness before clinical contracture develops [41,43,44]. Consensus efforts to standardize grading beyond the Baker scale are underway. Future trials may also test adjuvant anti-fibrotic therapies or therapeutic US for prevention or non-surgical management of early contracture, though these remain at preliminary stages.

**Figure 2:**
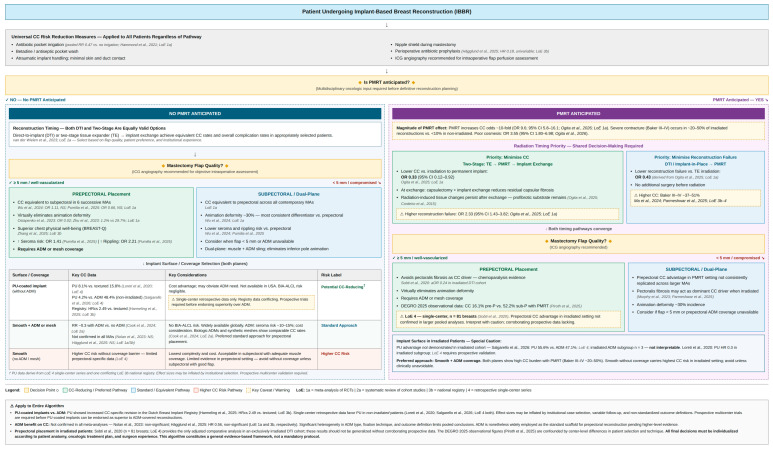
Proposed clinical decision framework for capsular contracture (CC) risk mitigation in implant-based breast reconstruction (IBBR). The algorithm stratifies decision-making by anticipated post-mastectomy radiotherapy (PMRT) status, radiation timing priority, mastectomy flap quality, implant placement plane, and implant surface/coverage selection. Evidence levels (LoE) are indicated at each decision node: 1a = meta-analysis of RCTs; 2a = systematic review of cohort studies; 3b = national registry; 4 = retrospective single-center series. Yellow diamonds = decision points; green boxes = CC-reducing/preferred pathways; blue boxes = standard/equivalent pathways; orange dashed boxes = key caveats/warnings. This algorithm is a general framework; effect estimates should be verified before clinical application. Individual patient anatomy, oncologic factors, and surgeon experience must guide all final decisions. This framework is intended as a pragmatic, conceptual proposal to guide individualized decision-making rather than a prescriptive protocol, given the predominantly retrospective and heterogeneous nature of the underlying evidence. Abbreviations: IBBR = implant-based breast reconstruction; CC = capsular contracture; PMRT = postmastectomy radiation therapy; TE = tissue expander; DTI = direct-to-implant; ADM = acellular dermal matrix; PU = polyurethane; ICG = indocyanine green; MA = meta-analysis; OR = odds ratio; RR = relative risk; HR = hazard ratio; HRcs = cause-specific hazard ratio; aOR = adjusted odds ratio; NS = non-significant; BIA-ALCL = breast implant–associated anaplastic large-cell lymphoma; LoE = level of evidence; RCT = randomised controlled trial; DEGRO = Deutsche Gesellschaft für Radioonkologie (German Society for Radiation Oncology); pre-P = prepectoral; sub-P = subpectoral. N.B.: Illustrated by our graphic designer, Marius Filip. [5,7,10,11,12,13,14,17,18,19,20,38,53,54,63,64,67,69,70,76].

Refinements in surgical technique and patient selection are expected to improve outcomes. Ensuring well-vascularized mastectomy flaps is critical: techniques such as laser-assisted indocyanine green angiography can help surgeons assess flap perfusion intraoperatively, potentially reducing ischemia-related complications that contribute to CC formation. Personalized reconstructive planning—matching the approach (DTI vs. staged; prepectoral vs. subpectoral; with or without ADM or PU) to each patient’s risk profile, anatomy, and oncologic needs—will be essential. To this end, we propose a clinical decision framework (Figure 2) that integrates the evidence reviewed herein, stratifying decision-making by PMRT status, radiation timing priority, mastectomy flap quality, implant placement plane, and surface/coverage selection. Ongoing registries and long-term outcome studies will continue to clarify best practices, while integration of patient-reported outcomes (e.g., BREAST-Q) will ensure that interventions are evaluated not merely by complication rates but by meaningful quality-of-life metrics.

## 11. Conclusions

CC remains the most common long-term complication of IBBR, driven by a complex interplay of subclinical infection, host immune dysregulation, surgical factors, and—most potently—radiation-induced fibrosis. Contemporary evidence indicates that modern reconstructive approaches—whether prepectoral or subpectoral, DTI or staged—achieve broadly comparable CC rates in non-irradiated patients when performed with appropriate technique and patient selection. Implant surface type (smooth, textured, PU) does not appear to be a dominant determinant of contracture in the reconstructive setting, though PU-coated implants show promise as a cost-effective alternative to ADM-covered implants. The most impactful modifiable risk factor remains radiation: PMRT increases contracture risk approximately 5–10-fold, and the optimal timing of radiation relative to reconstruction remains a subject of active debate, with trade-offs between contracture severity and reconstruction survival.

This review has several limitations inherent to the narrative design. Study selection was guided by expert judgment rather than a predefined systematic protocol, introducing potential selection bias. No formal risk-of-bias assessment or quantitative pooling was performed, limiting our ability to statistically evaluate the quality and consistency of the included evidence. A further limitation is the substantial heterogeneity among included studies in outcome definitions and assessment methods. CC was variably reported as clinically graded Baker III–IV CC, revision-requiring CC, or unspecified “CC”, making direct cross-study comparison problematic. The inconsistent application of the Baker classification—itself shown to have poor interobserver reliability (Section 4.1)—introduces additional measurement noise. Furthermore, differences in follow-up duration, ADM utilization rates, radiation protocols, and implant surface types across study populations limit the comparability of reported effect sizes, even within meta-analyses. These heterogeneity concerns should be borne in mind when interpreting the summary estimates presented throughout this review. Additionally, the rapid pace of publication in this field means that relevant studies may have been published after our search window closed. The predominance of retrospective, single-institution data for several key comparisons (e.g., PU vs. ADM, radiation timing) limits the strength of conclusions that can be drawn from the current evidence base.

Standardization of diagnostic criteria, long-term prospective registries, and head-to-head comparative trials (particularly for PU vs. ADM and for radiation timing strategies) are urgently needed to advance evidence-based practice and improve outcomes for the growing population of women undergoing IBBR.

## Figures and Tables

**Table 1:** Modified Baker–Spear Classification of Capsular Contracture.

Grade	Clinical Findings	Description
IA	Soft, natural feel.	The augmented/reconstructed breast feels as soft as an unoperated breast; implant not palpable.
IB	Implant palpable, no firmness.	Breast slightly firmer; implant palpable but not visible; no deformity.
II	Mild firmness.	Breast somewhat firm; implant palpable and possibly visible; no symptoms.
III	Moderate firmness, deformity.	Breast firm; implant easily palpable and often visible; breast distortion present; may or may not have discomfort.
IV	Hard, painful, deformed.	Breast hard, tender, and cold to touch; marked distortion and displacement; pain common.

Adapted from Baker (1978) [39] and Spear & Baker (1995) [40]. Grades III and IV are generally considered clinically significant and may warrant surgical intervention.

**Table 2:** Summary of Implant Surface Types in Breast Reconstruction [9,10,32,53,54,55,56,57,58,59,60].

Surface Type ([Ra] Range)	Tissue–Implant Interaction	CC Rate in Reconstruction	Key Considerations
Smooth (<10 µm)	Polished shell; minimal tissue adherence. Fibroblasts align parallel to implant surface in planar arrangement, facilitating circumferential contraction.	• OR 0.99 vs. textured (*p* = 0.97) [9]; • 4.7% CC rev. at 4 yrs (*n* = 494) [10].	• Most commonly used in current reconstructive practice globally. • No BIA-ALCL/SCC risk. • ↑ implant mobility (+/− ↑ rippling risk). • In augmentation, smooth implants carry significantly ↑ CC rates (OR 2.80 vs. textured) [55]; yet not in reconstruction, likely due to concurrent ADM use and mastectomy flap biology.
Microtxd. (10–50 µm)	Fine surface irregularities; moderate tissue ingrowth. Disrupts planar fibroblast alignment while limiting bacterial colonization surface area.	• Limited reconstruction-specific data: MAs—pooled with macrotxd. • May have ↓ CC rates vs. macrotxd [56,57].	• Intermediate profile—smooth vs. macrotxd. • BIA-ALCL risk substantially ↓ vs. macrotxd.; rare case reports only. • Balances CC reduction with improved safety.
Macrotxd. (>50 µm)	Deep, aggressive surface architecture; strong tissue ingrowth via mechanical interlock. Disrupts circumferential collagen orientation, but ↑ surface area = ↑ biofilm vulnerability.	• 2.4% CC rev. at 4 yrs (*n* = 3072) [10]; • 6.4% (shaped/textured) vs. 5.3% (round/smooth), RR 0.83, NS [58].	• Highest BIA-ALCL risk: 1:355–1:559 (MSKCC) [59]; all confirmed cases had textured implant history. • Also linked to BIA-SCC. • Allergan BIOCELL recalled globally 2019; declining use. • Strong tissue fixation; prevents rotation of anatomical implants.
Nanotextured (~4–6 µm)	Fine-scale surface nanopatterning; promotes controlled cellular adhesion without deep tissue ingrowth. Intermediate interaction between smooth and conventional texturing.	• Insufficient reconstruction-specific data. • Early series: ↓ CC with nanotextured TEs vs. smooth [60].	• Emerging technology; designed to balance CC reduction with BIA-ALCL safety. • No BIA-ALCL cases reported to date. • Lacking long-term validation in reconstruction cohorts. • Early adoption phase with ↑ initial complication rate attributed to learning curve [32].
PU-coated (N/A; foam coating)	PU foam gradually degrades in vivo over 1–2 years, promoting disorganized collagen deposition. Creates “double capsule” (inner PU remnant + outer fibrotic layer), preventing circumferential alignment.	• PU 8.1% vs. TE 15.8% (*p* = 0.009); HR 0.3 favoring PU [53]; with PMRT: 16.7% vs. 62.5% [53]. • 5.1% CC rev. at 4 yrs (*n* = 430) [10]. • PU 24.3% vs. ADM + TE 47.7% (Baker III–IV at 5 yrs.; *p* < 0.001) [54].	• Strong tissue fixation; ↓ seroma vs. ADM (2.9% vs. 33.8%) [54]. • May obviate ADM need; cost advantage. • Not available in USA. • Conflicting DBIR registry [10] vs. single-center data. • BIA-ALCL risk negligible. • 2,4-TDA degradation product—theoretical carcinogen; no clinical evidence of harm to date (see Appendix A Table A1).

↑ = increased/higher; ↓ = decreased/lower; +/− = with or without; µm = micrometer(s); *n* = number of patients/implants; *p* = *p*-value. Ra = roughness average (surface roughness parameter); CC = capsular contracture; rev. = revision; yr(s) = year(s); OR = odds ratio; RR = relative risk; HR = hazard ratio; NS = non-significant; BIA-ALCL = breast implant–associated anaplastic large-cell lymphoma; BIA-SCC = breast implant–associated squamous cell carcinoma; ADM = acellular dermal matrix; TE = tissue expander; PU = polyurethane; N/A = not applicable; PMRT = postmastectomy radiotherapy; Microtxd. = microtextured; Macrotxd. = macrotextured; MAs = meta-analyses; MSKCC = Memorial Sloan Kettering Cancer Center; DBIR = Dutch Breast Implant Registry; 2,4-TDA = 2,4-toluenediamine. Revision-based CC rates (Harmeling registry [10]) capture only contractures requiring surgical intervention (CC rev.) and are not directly comparable to clinically graded Baker III–IV rates. Detailed BIA-ALCL/BIA-SCC risk estimates and 2,4-TDA polymer degradation data are provided in Appendix A Table A1.

**Table 3:** Comparative Studies of Polyurethane-Coated Implants in Breast Reconstruction.

Study	Design (N; Follow-Up)	Comparator	Plane	PMRT Rate	CC Rate: PU vs. Comparator	Key Findings	Limitations
Loreti et al. 2020 [53]	Retrospective, single-center; 358 breasts DTI; median 2.3-year follow-up.	Textured	Mixed (sub-P + pre-P)	NR (high—strongest predictor).	Overall, Baker III–IV (clinical): PU (8.1%) vs. Textured (15.8%); *p* = 0.009.	PU HR 0.3 vs. textured (*p* = 0.003); PMRT strongest predictor (HR 12.5); PU advantage amplified in irradiated patients.	LoE 4. PMRT rate NR. Mixed placement planes. No ADM comparator arm.
Salgarello et al. 2025 [54]	Retrospective, single-center, 135 breasts DTI; 5-year follow-up.	ADM + Textured	Pre-P (both groups)	26.7% overall	Overall, Baker III–IV (clinical): PU—24.3% vs. ADM—47.7%; *p* < 0.001.	PU: lower seroma (2.9% vs. 33.8%, *p* < 0.001); superior symmetry and global aesthetic scores. ADM: lower rippling and implant visibility.	LoE 4. Non-randomized.
					Non-irradiated: 4.2% vs. 48.4%; Irradiated: 55.6% vs. 47.1%.	PU advantage driven entirely by non-irradiated subgroup; no meaningful difference in irradiated patients.	Very small irradiated subgroup; conclusions on irradiated patients are unreliable.
Harmeling et al. 2025 [10] *(DBIR)*	National registry, 3996 implants; Post-mastectomy reconstructions; 4-year follow-up.	Textured (mainly) + Smooth	Mixed (sub-P + pre-P)	8.6% (low)	Revision-requiring CC only: PU—5.1% vs. Textured—2.4% vs. Smooth—4.7%.	Overall adjusted: HRcs = 1.08 (95% CI 0.51–2.29, NS). CC-specific subgroup: HRcs = 2.49 (PU vs. textured, *p* < 0.05).	LoE 3b. Captures only revision-requiring CC—substantially underestimates true Baker III–IV incidence. Low PMRT rate (8.6%) limits applicability to oncologic population. Macrotextured device dominant comparator in NL.
Pompei et al. 2017 [49]	Retrospective, single-center, 228 breasts; Two-stage PU reconstructions; 9-year follow-up.	None (PU-only series)	NR	NR	Baker III–IV (clinical): 1.8% (PU only).	Lowest reported CC rate for any PU series. Long follow-up (9 years) is a methodological strength.	LoE 4. No comparator arm; data are descriptive only. Two-stage approach may contribute to low CC rate independently of surface type.

Studies are organized by comparator type and evidence level. Capsular contracture (CC) outcome definitions differ critically across studies and are noted for each entry: Loreti, Salgarello, and Pompei report clinically graded Baker III–IV contractures, whereas Harmeling reports only contractures requiring revision surgery, which captures only the most severe end of the clinical spectrum and substantially underestimates true CC incidence. The Salgarello entry is stratified by postmastectomy radiotherapy (PMRT) status, as the polyurethane (PU) advantage was confined to non-irradiated patients (PU 4.2% vs. ADM 48.4%), while irradiated subgroups showed no meaningful difference (note: very small ADM subgroup, *n* = 3). Comparator surfaces, placement planes, and PMRT rates vary substantially across studies, precluding direct cross-study comparison. All studies except Harmeling are single-center retrospective designs (LoE 4); Harmeling is a national registry study (LoE 3b). No prospective randomized data comparing PU-coated implants with other surface types in breast reconstruction currently exist. CC = capsular contracture; PU = polyurethane; ADM = acellular dermal matrix; HR = hazard ratio; HRcs = cause-specific hazard ratio; DBIR = Dutch Breast Implant Registry; DTI = direct-to-implant; PMRT = postmastectomy radiotherapy; NR = not reported; NS = non-significant; NL = Netherlands; pre-P = prepectoral; sub-P = subpectoral; LoE = level of evidence.

**Table 4:** Summary of Key Meta-Analyses and Registry Studies on Risk Factors for Capsular Contracture in Implant-Based Breast Reconstruction.

Study	Comparison	Effect Size (OR/RR/HR)	Key Finding
***Implant Surface and Fill***			
Christodoulou 2024 [9]	Smooth vs. textured (23 studies)	OR 0.99 *p* = 0.97	No CC difference between implant surfaces in the reconstruction setting; parity may be confounded by concurrent ADM use.
	Saline vs. silicone (23 studies)	OR 0.19 95% CI: 0.08–0.43	81% lower CC odds with saline; based on only 2 reconstruction studies with limited power. Not replicated in augmentation data *.
Barnsley et al. 2006 [33]	Textured vs. smooth (augmentation; meta-analysis of RCTs)	Pooled OR 0.19; 95% CI 0.07–0.52	Textured implants significantly reduce CC vs. smooth in augmentation; effect not replicated in reconstruction setting [9], likely confounded by ADM use. Included for mechanistic context only †.
***Implant Placement Plane***			
Li et al. 2020 [68]	Prepectoral vs. subpectoral (15 studies; 1868 patients)	OR 0.45 95% CI: 0.27–0.73	Earliest MA to report significant CC advantage for prepectoral; not replicated in subsequent larger analyses.
Ostapenko et al. 2023 [11]	Prepectoral vs. subpectoral (15 studies; 3101 patients)	OR 0.54 95% CI: 0.32–0.92	PP significantly lower CC (10 studies; I^2^ = 53%); also, lower prosthesis failure (OR 0.61) and animation deformity (OR 0.02). Mean follow-up 19 months; no ADM stratification.
Christodoulou 2024 [9]	Subpectoral vs. prepectoral (16 studies)	OR 1.21 95% CI: 0.75–1.95	No CC difference by placement plane; ADM subgroup analyses also NS in all strata.
Wu et al. 2024 [12]	Prepectoral vs. subpectoral (40 studies; 12,943 breasts)	OR 1.11 95% CI: 0.65–1.92	No CC difference; PP associated with higher seroma (OR 1.55) and rippling (OR 2.39) but lower animation deformity (OR 0.37).
Zhu et al. 2023 [13]	Prepectoral vs. partial subpectoral (ADM) (10 studies; 2667 reconstructions)	RR 0.939 95% CI: 0.68–1.30	Equivalent CC rates when both planes use ADM; animation deformity dramatically lower with prepectoral (1.2% vs. 29.7%).
Pumilia et al. 2025 [69]	Prepectoral vs. submuscular (47 studies; 12,074 breasts)	OR 0.66 95% CI: 0.43–1.01	Largest MA; NS trend favoring PP (*p* = 0.06). PP had higher seroma (OR 1.41) and rippling (OR 2.21) but markedly lower animation deformity (OR 0.09).
Sobti et al. 2020 [64]	Prepectoral vs. subpectoral DTI (Single institution; 47 pts, 81 breasts; irradiated cohort)	aOR 0.24 95% CI: 0.08–0.64	PP significantly reduced CC in irradiated DTI cohort (30% vs. 52%); ADM type NS (OR 1.08). Muscle fibrosis identified as primary contracture driver via chemoparalysis evidence.
***Radiotherapy***			
Ogita et al. 2025 [17]	PMRT to TE vs. PMRT to implant (11 studies; 1628 cases)	OR 0.33 95% CI: 0.12–0.92	TE irradiation significantly lowers CC. The trade-off is higher reconstruction failure (OR 2.33, 95% CI: 1.43–3.82). No difference in overall major complications
Ogita et al. 2026 [18]	PMRT vs. no PMRT (Updated meta-analysis)	OR 9.6 95% CI: 5.8–16.1	PMRT increases CC risk nearly 10-fold; poor cosmesis OR 3.55 (95% CI: 1.80–6.98). Strongest single risk factor identified herein.
Parmeshwar et al. 2025 [70]	PMRT vs. no PMRT (DTI prepectoral cohort)	OR 8.9 *p* < 0.001	Confirms high CC risk with RT in the DTI prepectoral setting.
***Device Characteristics and ADM***			
Hägglund et al. 2025 [7]	Permanent TE vs. fixed-volume implant (1095 breasts; Swedish registry)	HR 19.3 95% CI: 3.9–95.4	Permanent TE (combined expander-implant) dramatically increases CC; device type outweighs reconstruction timing or placement plane effects.
Cook et al. 2024 [63]	ADM vs. no ADM (Prepectoral systematic review)	RR 0.3	ADM may reduce CC by 60–70% in prepectoral reconstruction; not confirmed in all MAs **

Studies are organized by thematic domain (implant surface and fill, placement plane, radiotherapy, device characteristics and ADM) and listed chronologically within each domain to illustrate the evolution of evidence over successive pooled analyses. Effect sizes are reported as published in each source study; note that the direction of comparison varies (e.g., prepectoral vs. subpectoral in some analyses, subpectoral vs. prepectoral in others), and the reader should attend to which group serves as reference when interpreting odds ratios. The table includes predominantly meta-analyses and national registry studies, but also one single-institution comparative study (Sobti et al. [64]) reporting the only available adjusted analysis of placement plane in an exclusively irradiated cohort; this entry should be interpreted with appropriate caution given its small sample size (*n* = 81 breasts). Superscript annotations denote instances where findings from the cited study were not replicated in other pooled analyses: * indicates that the saline advantage over silicone (OR 0.19) derives from only two reconstruction studies totaling 132 cases and was not significant in augmentation data (Haas 2025 [55]: OR 0.39, *p* = 0.52); ** indicates that the ADM protective effect reported by Cook et al. was not confirmed in all meta-analyses (Hägglund 2025 [7]: HR 0.56, *p* = 0.54; Nolan 2023 [14]: NS). † Augmentation-specific meta-analysis included for comparison; findings are not directly applicable to the post-mastectomy reconstruction setting. Where 95% confidence intervals are available, they are provided alongside point estimates; entries without CIs reflect the reporting in the original source. CC = capsular contracture; OR = odds ratio; RR = relative risk; HR = hazard ratio; aOR = adjusted odds ratio; CI = confidence interval; TE = tissue expander; DTI = direct-to-implant; MA(s) = meta-analysis(es); PP = prepectoral; PMRT = postmastectomy radiotherapy; ADM = acellular dermal matrix; NS = non-significant; RT = radiotherapy; I^2^ = heterogeneity statistic; pts = patients.

## Data Availability

Data available on request.

## Abbreviations

The following abbreviations are used in this manuscript:

**2,4-TDA**	2,4-Toluenediamine
**ADM**	Acellular Dermal Matrix
**BIA-ALCL**	Breast Implant–Associated Anaplastic Large-Cell Lymphoma
**BIA-SCC**	Breast Implant–Associated Squamous Cell Carcinoma
**BMI**	Body Mass Index
**BREAST-Q**	Breast Reconstruction Evaluation and Aesthetics Satisfaction Tool–Questionnaire
**BTX-A**	Botulinum Toxin A
**CC**	Capsular Contracture
**CI**	Confidence Interval
**COX-2**	Cyclooxygenase-2
**CT**	Computed Tomography
**DBIR**	Dutch Breast Implant Registry
**DEGRO**	Deutsche Gesellschaft für Radioonkologie (German Society for Radiation Oncology)
**DTI**	Direct-to-Implant
**FABREC**	Fractionation After Breast Reconstruction (trial)
**FBR**	Foreign Body Response
**FDA**	U.S. Food and Drug Administration
**Gy**	Gray (unit of absorbed radiation dose)
**HR**	Hazard Ratio
**HRcs**	Cause-Specific Hazard Ratio
**IBBR**	Implant-Based Breast Reconstruction
**ICG**	Indocyanine Green
**IL-6**	Interleukin-6
**IL-17**	Interleukin-17
**IMF**	Inframammary Fold
**IMRT**	Intensity-Modulated Radiation Therapy
**ISO**	International Organization for Standardization
**LoE**	Level of evidence
**MA**	Meta-Analysis
**MPC**	2-Methacryloyloxyethyl phosphorylcholine
**MRI**	Magnetic Resonance Imaging
**MSKCC**	Memorial Sloan Kettering Cancer Center
**NS**	Non-Significant
**NSQIP**	National Surgical Quality Improvement Program
**OR**	Odds Ratio
**PCR**	Polymerase Chain Reaction
**PDMS**	Polydimethylsiloxane
**PMRT**	Postmastectomy Radiation Therapy
**PU**	Polyurethane
**Ra**	Roughness Average (surface roughness parameter)
**RCT**	Randomized Controlled Trial
**ROS**	Reactive Oxygen Species
**RR**	Relative Risk
**RT**	Radiotherapy
**TE**	Tissue Expander
**TGF-β/-β1**	Transforming Growth Factor–Beta / –Beta 1
**Th17**	T Helper 17 (cell subset)
**Treg**	Regulatory T Cell
**US**	Ultrasound
**VMAT**	Volumetric Modulated Arc Therapy
**α-SMA**	α-Smooth Muscle Actin
**κ**	Kappa (Cohen’s κ; measure of interobserver agreement)

## Appendix A. Supplementary Data

**Table A1:** Neoplasia Risk Estimates (BIA-ALCL and/or BIA-SCC) by Implant Surface Type, and Possible Carcinogens Associated with PU-Coated Implants (2,4-TDA Degradation Data).

Topic	Surface Type	Data/Estimate	Source/Notes
BIA-ALCL risk	Macrotextured	1:355–1:559 lifetime risk per implanted patient (MSKCC series). All confirmed BIA-ALCL cases globally have had a history of textured implant exposure. Risk is highest with Allergan BIOCELL (recalled globally July 2019) and Siltex devices.	[50]; FDA/WHO surveillance data.
	Microtextured	Substantially lower than macrotextured; rare case reports only, no population-level incidence estimate established.	FDA device surveillance; [50].
	Smooth	No confirmed cases in patients with exclusive smooth implant history.	FDA/WHO; [50,52].
	Nanotextured (~4–6 µm)	No confirmed cases to date. Long-term surveillance data lacking; cannot exclude very low residual risk.	[60]; manufacturer registry data.
	PU-coated (foam)	Negligible; PU foam surface does not generate the type of particulate debris and chronic T-cell stimulation implicated in BIA-ALCL pathogenesis. No confirmed cases reported.	[48,49]; mechanistic review.
BIA-SCC	Macrotextured (and textured generally)	Rare but increasingly recognized; associated with chronic seroma and implant texturing. Exact incidence unknown; case series and systematic reviews ongoing.	[51].
	Smooth/PU-coated	Case reports exist but no established causal link to surface type confirmed to date.	[51].
2,4-TDA degradation (PU implants)	PU-coated	PU foam degrades in vivo over 1–2 years, releasing 2,4-toluenediamine (2,4-TDA) as a breakdown product. 2,4-TDA is classified as a probable carcinogen (IARC Group 2B) based on animal data. Urine levels of 2,4-TDA have been detected in patients with PU implants, but concentrations are extremely low (parts per trillion range) and no clinical cases of carcinogenesis attributable to PU implant degradation have been reported in over three decades of use (predominantly in Europe and South America). PU implants remain unavailable in the USA largely due to this theoretical concern.	[48,49]; European regulatory assessments.

BIA-ALCL = breast implant–associated anaplastic large-cell lymphoma; BIA-SCC = breast implant–associated squamous cell carcinoma; PU = polyurethane; 2,4-TDA = 2,4-toluenediamine; MSKCC = Memorial Sloan Kettering Cancer Center; IARC = International Agency for Research on Cancer; FDA = U.S. Food and Drug Administration; WHO = World Health Organization. All citation numbers refer to the main manuscript reference list.
